# Dietary and Microbial Metabolites in the Regulation of Host Immunity

**DOI:** 10.3389/fmicb.2017.02171

**Published:** 2017-11-07

**Authors:** Naoko Shibata, Jun Kunisawa, Hiroshi Kiyono

**Affiliations:** ^1^Division of Mucosal Immunology, Department of Microbiology and Immunology and International Research and Development Center for Mucosal Vaccines, The Institute of Medical Science, The University of Tokyo, Tokyo, Japan; ^2^Department of Mucosal Immunology, School of Medicine, Chiba University, Chiba, Japan; ^3^Laboratory of Vaccine Materials and Laboratory of Gut Environmental System, National Institutes of Biomedical Innovation, Health and Nutrition, Osaka, Japan; ^4^Graduate School of Medicine, Graduate School of Pharmaceutical Sciences, Graduate School of Dentistry, Osaka University, Osaka, Japan; ^5^Department of Microbiology and Infectious Diseases, Kobe University Graduate School of Medicine, Kobe, Japan; ^6^Department of Immunology, Graduate School of Medicine, Chiba University, Chiba, Japan

**Keywords:** microbiome, metabolite, fatty acid, vitamin

## Abstract

Mucosal surfaces in the body, especially the intestine, are constantly exposed to trillions of microbiomes. Accumulating evidence has revealed that changes in the composition of the gut microbiome, especially that of the commensal bacteria population, are frequently associated with immunologic disorders. These changes coincide with changes in the production of certain dietary metabolites. Recent studies have uncovered the molecular and cellular mechanisms underlying the relationships among diet, commensal bacteria, and the host immune system. In this review, we describe how dietary and microbial metabolites modulate host immunity.

## Introduction

Mucosal surfaces, especially that of the gastrointestinal tract, are constantly exposed to a wide variety of antigens including trillions of bacteria and fungi, as well as dietary components and their metabolites. The host immune system discriminates between harmful and beneficial antigens, simultaneously inducing immune responses to exclude harmful antigens while tolerating beneficial antigens to establish appropriate homeostatic conditions in the gut.

It has long been recognized that commensal bacteria regulate host responses, including immunity, but, due to the difficulty of culturing the intestinal microbiome, it has been challenging to obtain information. In addition, because dietary and microbial metabolites are generated in a complex network that includes the diet, host, and intestinal microbiome, it is difficult to analyze such metabolites. However, recent advances in genome-based analysis of bacteria has enabled direct analysis of the intestinal microbiome without culturing; this analysis has revealed that changes in the composition of the gut microbiome are associated with immunologic disorders and diseases (Browne et al., 2017). In addition, advances in high throughput metabolomics have allowed immunologists to investigate metabolites generated in the intestine and to discover that these changes in the microbiome composition coincide with changes in either useful or harmful metabolites involved in the regulation of immune responses. For example, commensal bacteria are involved in the extraction, synthesis, and absorption of many nutrients and metabolites including short- and LCFAs, and vitamins (Hirata and Kunisawa, 2017).

In this review, we describe recent findings regarding the role of the gut microbiome–diet interaction in the generation of immunologically active metabolites.

## Long-Chain Fatty Acids in the Control of Immune Responses

**FIGURE 1:**
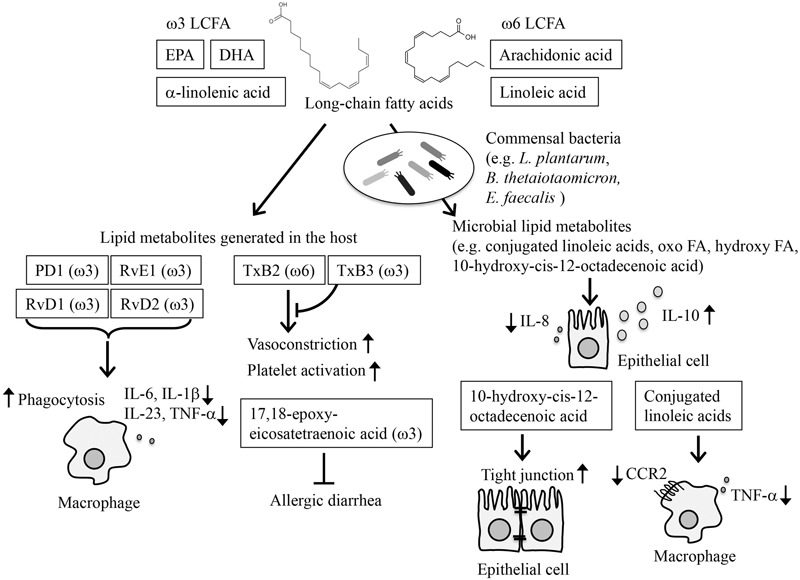
Modification of immune responses by lipid metabolites. LCFAs are converted to bioactive lipid metabolites by host enzymes and commensal bacteria (e.g., *L. plantarum, B. thetaiotaomicron, E. faecalis*), and participate in the regulation of immune responses. Among LCFAs, ω3 LCFAs, such as EPA, DHA, and α-linolenic acid, and ω6 LCFA, such as AA and linoleic acid, are essential FAs. PD1, RvE1, RvD1, and RvD2, which are derived from EPA/DHA, activate inflammation resolution programs by modifying monocyte functions, such as inhibiting the production of inflammatory cytokines while enhancing phagocytosis. TxB3, derived from EPA, competitively inhibits the vasoconstriction and platelet activation induced by TxB2, which is derived from AA. 17,18-epoxy-eicosatetraenoic acid is derived from ω3 LCFA and participates in the amelioration of allergic diarrhea. Microbial lipid metabolites inhibit IL-8 production and activate IL-10 production from IECs. Conjugated linoleic acids inhibit the inflammatory responses of macrophages. 10-hydroxy-cis-12-octadecenoic acid, generated by commensal bacteria, enhances the tight junctions on IECs.

Fatty acids are an essential nutrient that is mainly obtained from the diet. Generally, dietary oils are composed of various LCFAs. LCFAs primarily serve as a source of energy and membrane components, but they are also actively involved in the regulation of immune responses by being metabolized into lipid metabolites (**Figure 1**) (Hirata and Kunisawa, 2017).

Studies to date have mainly focused on the lipid metabolites generated after absorption into the body. Among LCFAs, ω3 and ω6 LCFAs are essential FAs, meaning that they are not generated by mammals, including humans (Hirata and Kunisawa, 2017). These essential FAs are metabolized into bioactive lipid mediators through reactions that are mediated by several series of oxidative enzymes, such as cyclooxygenases, lipoxygenases, and cytochrome P450 monooxygenases (Arita, 2012). It is thought that ω3 LCFAs have anti-allergic and anti-inflammatory properties, whereas ω6 LCFAs have pro-inflammatory properties (Arita, 2012; Serhan, 2017). AA and linoleic acid are the major ω6 LCFA, whereas α-linolenic acid abundantly present in linseed or perilla oil, EPA and DHA abundantly present in fish oils are typical ω3 LCFAs. TX B_2_, an AA derivative, is a potent vasoconstrictor and platelet activator, whereas TXB_3_, an EPA derivative, has barely any physiological effects. Dietary supplementation with EPA has been shown to reduce TXB_2_ levels in plasma and cellular fatty acid and to induce a shift to less reactive platelets and to reduce blood pressure in response to pressor hormones (Weber et al., 1986). In another study, dietary supplementation with ω3 LCFAs suppressed the ability of monocytes to synthesize inflammatory cytokines, such as interleukin-1 (IL-1) and TNF, which was accompanied by a decreased ratio of AA to EPA in the membrane phospholipids of the monocytes (Endres et al., 1989). Since the conversion of ω3 and ω6 LCFAs to bioactive lipid mediators shares the same series of enzymes, competition exists between AA and EPA/DHA for metabolism. This competition is one of the mechanisms through which EPA inhibits the inflammatory properties of AA.

In addition to the competition between ω3 and ω6 LCFAs, some lipid metabolites of ω3 LCFAs exhibit anti-inflammatory or anti-allergy effects (Arita, 2012; Kunisawa et al., 2015a). E-series Rv are derived from EPA, whereas D-series Rv and Protectin D1 (PD1) are derived from DHA. RvD1, RvD2, RvE1, and PD1 activate inflammation resolution programs by reducing levels of pro-inflammatory cytokines, including IL-6, IL-1β, IL-23, and TNFα, and neutrophil influx, and by promoting macrophage phagocytosis of apoptotic cells and inflammatory debris (Schwab et al., 2007; Spite et al., 2009; Chiang et al., 2012).

In addition to these studies, we previously reported that allergic diarrhea was ameliorated when mice were maintained on linseed oil rich in ω3 α-linolenic acid (Kunisawa et al., 2015a). The α-linolenic acid is metabolized to eicosapentaenoic acid, levels of which also increased in the intestine of mice maintained on linseed oil. When we performed lipidomics analysis, we identified 17,18-epoxy-eicosatetraenoic acid as an anti-allergic lipid metabolite derived from eicosapentaenoic acid (Kunisawa et al., 2015a). Given that synthetic 17,18-epoxy-eicosatetraenoic acid administration is sufficient to inhibit allergic diarrhea, this lipid metabolite appears to be effective in the control of intestinal allergy.

Although these lipid metabolites seem to be generated in the body, several lines of evidence indicate that commensal bacteria also express enzymes that participate in LCFA metabolism (**Figure 1**). Indeed, germ-free animals exhibit alterations of composition in their lipid metabolites (Claus et al., 2008; Martin et al., 2009; Chiang et al., 2012). In this regard, we identified conjugated linoleic acids, oxo FAs, and hydroxy FAs as microbial lipid metabolites (Kishino et al., 2013). These metabolites are formed in the intestine through the action of commensal bacteria, especially *Lactobacillus plantarum* (Kishino et al., 2013). Indeed, these lipid metabolites are abundant in the intestinal lumen of specific pathogen-free mice but are scarce in germ-free mice (Kishino et al., 2013). Of note, these lipid metabolites are detected not only in the intestinal lumen but also in the serum, suggesting that these microbial lipid metabolites act both in the intestinal lumen and elsewhere in the body.

Administration of 10-hydroxy-cis-12-octadecenoic acid, one of the microbial lipid metabolites, has been reported to ameliorate experimental colitis by enhancing tight junctions on epithelial cells. Signaling though LCFA receptors is mediated by GPR40, which suppresses TNFR2 gene expression and NF-κB via the MEK-ERK pathway (Miyamoto et al., 2015). LCFAs are also recognized by PPARs, which also modulate immune reactions and allergic diseases (Ricote et al., 1998; Fukui et al., 2009). For example, it was reported that probiotic bacteria in the intestine produce conjugated linoleic acids, which target PPAR-γ in macrophages to suppress the inflammatory response (Bassaganya-Riera et al., 2012). In other studies, *Bacteroides thetaiotaomicron* activated PPAR-γ, which decreased NF-κB–dependent IL-8 production (Kelly et al., 2004), and *Enterococcus faecalis* activated PPAR-γ1 in IECs thereby increasing the production of IL-10 in human newborn babies (Are et al., 2008). Although it remains unclear which types of lipids act as ligands for these PPRA receptors, these microbial lipids and lipid metabolites may prevent allergy and inflammation.

As mentioned before, RvD1, RvD2, RvE1, and PD1 participate in the activation of inflammation resolution programs (Schwab et al., 2007; Spite et al., 2009; Chiang et al., 2012). It is important to note that commensal bacteria are also involved in this process (Chiang et al., 2012). Given that germ-free mice have increased levels of endogenous RvD1 and PD1 in their colon (Chiang et al., 2012), these reports suggest that microbial suppression of RvD1 and PD1 production may be involved in the regulation of the host immune defense against invading pathogens.

## Short-Chain Fatty Acids in the Control of Immune Responses

**FIGURE 2:**
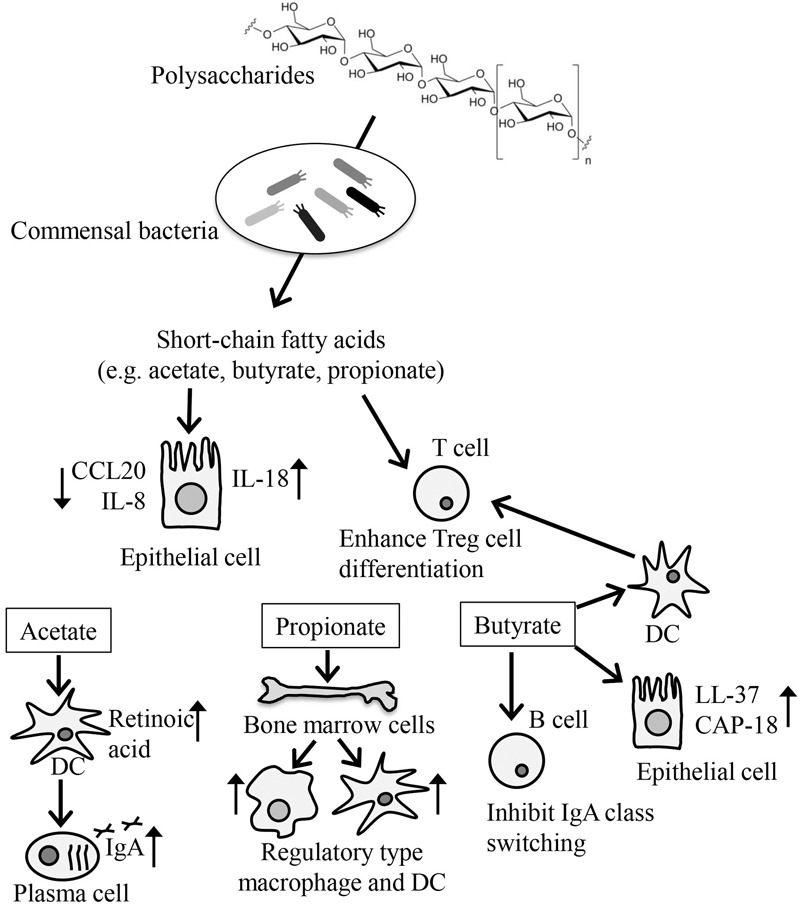
Modification of immune responses by SCFAs produced by microbial fermentation. SCFAs are produced by bacterial fermentation of dietary indigestible polysaccharides and participate in the regulation of energy metabolism, cellular function, and differentiation. SCFAs, such as acetate, butyrate, and propionate, are taken up by IECs, where they inhibit the production of inflammatory cytokines such as CCL20 and IL-8 and enhance the production of IL-18, which is involved in IEC integrity. Butyrate participates in the production of antimicrobial peptides such as LL-37 and CAP-18. SCFAs also directly enhance the differentiation of Treg cells. Butyrate acts on DCs to enhance DC-induced Treg cell differentiation. Acetate enhances the production of retinoic acids from DCs, which promote IgA production, whereas butyrate inhibits the IgA class switching of B cells. Propionate enhances the generation of regulatory type of macrophages and DCs from bone marrow cells.

Short-chain fatty acids are present at high concentrations in the intestine as bacterial fermentation products of dietary indigestible polysaccharides such as cellulose (**Figure 2**) (Wong et al., 2006). Because mammals lack the enzymes to degrade polysaccharides, germ-free mice exhibit remarkably decreased amounts of SCFAs and increased amounts of indigestible oligosaccharide, a bacterial fermentation substrate (Hoverstad and Midtvedt, 1986).

Among SCFAs, acetate, propionate, and butyrate have been well studied. Accumulated evidence indicated that these SCFAs modify several cellular processes including gene expression, chemotaxis, differentiation, proliferation, and apoptosis, which affect various biological responses including the immune response (**Figure 2**) (Correa et al., 2016). Generally, SCFAs are recognized on the cell surface by G-protein coupled receptors (GPRs) such as GPR41, GPR43, GPR109A, and Olfr78 (Bolognini et al., 2016). SCFAs are also transported by monocarboxylate transporter-1 and the sodium-dependent monocarboxylate transporter-1 and by passive diffusion across the plasma membrane into the cytoplasm (Thwaites and Anderson, 2007).

Because SCFAs are found at high concentrations in the intestine, they are in direct contact with the IECs. IECs take up SCFAs through both passive and active mechanisms into their cytosol, where the SCFAs, especially butyrate, are used as a source of ATP for energy metabolism (den Besten et al., 2013). In addition to their role in energy metabolism, SCFAs enhance some immune surveillance functions of IECs by increasing the expression of certain antimicrobial peptides; for example, butyrate increases the expression of LL-37 and CAP-18 (Raqib et al., 2006), and modulating cytokine (CCL20 and IL-8) production (Iraporda et al., 2015). In addition, the activation of GPR43 and GPR109a by SCFAs in IECs reportedly resulted in an increase in the production of IL-18, a cytokine involved in the maintenance of epithelial integrity (Singh et al., 2014; Macia et al., 2015).

Short-chain fatty acids are also known to enhance the induction of Treg cells in the intestine. A key molecule involved in this event is the butyrate receptor GPR109a expressed on DCs. Indeed, in one study, GPR109a-deficient mice were found to have reduced numbers of Treg cells (Singh et al., 2014). In another study, SCFAs were found to directly affect the preferential differentiation of T cells to Treg cells with concurrent enhancement of histone H3 acetylation in the Foxp3 locus by butyrate, or the GPR43-mediated pathway (Furusawa et al., 2013; Smith et al., 2013). Consistent with these findings, mice lacking either GPR43 or GPR109a show exacerbated symptoms in food-allergy models (Tan et al., 2016). In addition to GPR43 and GPR109a, Slc5a8, a Na^+^-coupled high-affinity transporter for SCFAs such as butyrate, plays an important role in the increased expression of indoleamine 2,3-dioxygenase 1 and aldehyde dehydrogenase in DCs, which results in the preferential induction of FoxP3^+^ Treg cells (Gurav et al., 2015).

Besides their effects in the intestine, SCFAs exhibit several anti-allergic properties after absorption into the body. For example, mice fed a high-fiber, but not mice fed a low-fiber, showed increased levels of SCFAs in the blood and were protected against allergic inflammation in the lung (Trompette et al., 2014). In this case, propionate enhanced the generation of macrophage and DC precursors from bone marrow cells, which is dependent on GPR41 but not GPR43. DCs and macrophages generated in this process are highly phagocytic but impaired in their ability to promote the effector function of Th2 cells, thereby blocking allergic responses (Trompette et al., 2014).

Short-chain fatty acid are also involved in the production of intestinal IgA. The acetate-GPR43 axis positively regulates this process. Indeed, supplementation of acetate promotes intestinal IgA production in a GPR43-dependent manner (Wu et al., 2017). As an underlying mechanism, it was demonstrated that acetate induces the expression of aldehyde dehydrogenase in DCs, which converts vitamin A to retinoic acid for the promotion of IgA production (Wu et al., 2017). In contrast to the positive effects of acetate on intestinal IgA production, butyrate has been reported to suppress class switching to IgA through the upregulation of miR-155, -181b, -361, -23b, -30a, and -125b in B cells, which silence AID and Blimp-1 (White et al., 2014). These findings collectively suggest that a balance among SCFAs controls the immunologic status quo in both humoral and cellular immunity.

Short-chain fatty acids regulate IEC functions controlled by TLRs. IECs express several TLRs, including TLR2/1, TLR4, TLR5, and TLR9, which mainly recognize bacterial lipoproteins, lipopolysaccharides, flagellin, and DNA, respectively (de Kivit et al., 2014). SCFAs have been reported to modify immune responses by altering TLR-induced inflammatory gene expression through the inhibition of histone deacetylases (Lin et al., 2015). For example, when IECs were incubated with butyrate or propionate and then stimulated with flagellin, a ligand for TLR5 (Oh et al., 2014), the expression levels of pro-inflammatory cytokines such as TNF-α were upregulated, whereas those of chemotactic chemokines, such as IL-8 and monocyte chemotactic protein-1, were downregulated (Lin et al., 2015). A similar regulation pathway, mediated by TLR ligands and SCFAs, has been observed in hematopoietic cells (Mirmonsef et al., 2012). Given that many TLR ligands are present in not only pathogenic but also commensal bacteria and that SCFAs are produced by these bacteria via fermentation, the combination of TLR ligands and SCFAs seems to be an essential component of one of the commensal-mediated immune regulation pathways. Of note, flagellin-initiated TLR5 stimulation plays essential roles in the induction of immune responses against trivalent inactivated influenza vaccine-induced antibody responses (Oh et al., 2014). These reports indicate that the effectiveness of vaccination varies depending on the balance between commensal bacteria carrying flagellins and commensal bacteria contributing to the synthesis of SCFAs.

## Vitamin B Family in the Control of Immune Responses

**FIGURE 3:**
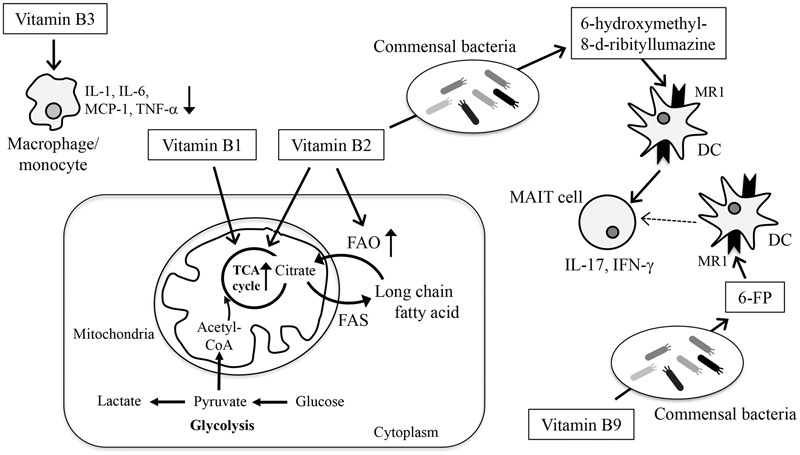
Modification of energy metabolism and immune responses by vitamins. Vitamins are involved in the maintenance of immunological homeostasis in the gut through the regulation of energy metabolism, cellular function, and differentiation. Glucose enters the cell and is metabolized to pyruvate, which is then converted to lactate (glycolysis) or enters the TCA cycle for energy generation. In the TCA cycle, citrate can be transferred out of the mitochondria into the cytoplasm, where it is used for fatty acid synthesis (FAS). Fatty acids can be degraded in the mitochondria and enter the TCA cycle to produce energy (FAO). Vitamins B1 and B2 act as cofactors for enzymes involved in the TCA cycle. Vitamin B2 also acts as a cofactor for enzymes involved in FAO. Vitamin B3 inhibits the production of inflammatory cytokines such as TNF-α, IL-6, IL-1, MCP-1 from macrophages and monocytes. Vitamins B2 and B9 are metabolized by commensal bacteria and converted to 6-hydroxymethyl-8-D-ribityllumazine and 6-FP, respectively. 6-hydroxymethyl-8-D-ribityllumazine activates MAIT cells, whereas 6-FP binds to MR1 without activating MAIT cells.

Mammals do not have biosynthetic pathways for vitamins and therefore must obtain vitamins externally. Of course, diets are a general source of vitamins, but commensal bacteria also produce vitamins and simultaneously consume dietary vitamins. Therefore, both diet and commensal bacteria determine the vitamin contents in the intestine (**Figure 3**).

Vitamins are required for the maintenance of many biological responses by acting as antioxidants, transcription factors, and cofactors for metabolic enzymes in the generation, conversion, and digestion of fatty acids, nucleotides, carbohydrate, and amino acids. Immune cells require all of these processes for their development, differentiation, and activation and therefore vitamin deficiency is frequently associated with increased risk of infectious, allergic, and inflammatory diseases (**Figure 3**) (Kunisawa and Kiyono, 2015; Suzuki and Kunisawa, 2015).

Vitamin B3 (nicotinic acid) supplementation acts on monocytes to dampen TLR2- and TLR4-induced release of inflammatory mediators such as TNF-α, IL-6, and monocyte chemotactic protein-1 (Digby et al., 2012). These effects are mediated by GPR109a, a vitamin B3 receptor expressed on monocytes (Digby et al., 2012). Vitamin B3 also reduces macrophage production of pro-inflammatory cytokines, including IL-1, IL-6, and TNF-α, in a murine model of atherosclerosis (Lipszyc et al., 2013). Collectively, these findings suggest that vitamin B3 exerts its anti-inflammatory properties by modulating immune cells.

The vitamin B complex also contributes to energy metabolism. Currently, the metabolic processes in immune cells are recognized as the emerging field of immunometabolism (Buck et al., 2017; Mills et al., 2017). The core function of metabolic pathways is the synthesis or degradation of sugars, fatty acids, nucleic acids, or proteins, coupled to the consumption or generation of ATP by oxidative phosphorylation or glycolysis. Changes in these metabolic pathways are frequently associated with immune cell functions such as cell proliferation and the production of cytokines, chemokines, and antibodies (Buck et al., 2017; Mills et al., 2017). Recent advances in our understanding of immunometabolism have revealed that quiescent or regulatory-type cells such as naïve T and B cells, Treg cells, and M2 macrophages use anabolic pathways for energy generation from the TCA cycle, such as FAO, whereas activated or inflammatory cells (e.g., Th1, Th2, Th17, IgA-producing plasma cells, M1 macrophages) use catabolic pathways and shift to glycolysis for energy generation (Buck et al., 2017; Kunisawa, 2017; Mills et al., 2017).

In metabolic energy pathways, vitamin B2 and its active forms (e.g., flavin adenine dinucleotide) function as cofactors for various enzymatic reactions such as the TCA cycle and FAO (Huskisson et al., 2007). FAO is necessary for the generation of acetyl-CoA, which enters the TCA cycle in mitochondria to produce energy. Studies using rodent models have shown that vitamin B2 deficiency reduces the activity of acyl-CoA dehydrogenases, which participate in the dehydrogenation step of FAO, and that vitamin B2 supplementation rescues the activity of these enzymes (Sakurai et al., 1982). As with vitamin B2, vitamin B1 (also known as thiamine) and its derivatives (e.g., thiamine pyrophosphate) acts as a cofactor for several enzymes such as pyruvate dehydrogenase and α-ketoglutarate dehydrogenase that are involved in TCA cycle (Frank et al., 2007). In agreement with the importance of energy metabolism in immune system, mice maintained on diets lacking the vitamin B complex show impaired immunity (Kunisawa et al., 2015b; Suzuki and Kunisawa, 2015; Hosomi and Kunisawa, 2017).

In addition to their direct effects on immune cells and energy metabolism, microbial metabolites of some B vitamins act as ligands for immune cells, especially mucosal-associated invariant T (MAIT) cells. Activated MAIT cells produce IL-17 and IFN-γ and help exclude infectious bacteria (Bourhis et al., 2013). Because MAIT cells are found in several inflammatory tissues in brain and kidney cancer patients, accompanied by reduced numbers of MAIT cells in the circulating blood, it is thought that MAIT cells infiltrate the inflammatory tissues and contribute to inflammatory disease (Bourhis et al., 2013). MAIT cells are innate-like T cells that recognize MHC-like protein 1 (MR1)-restricted presentation. Intriguingly, the microbial metabolite of vitamin B2 (riboflavin) 6-hydroxymethyl-8-D-ribityllumazine has been shown to activate MAIT cells (Kjer-Nielsen et al., 2012; Patel et al., 2013). Similarly, a microbial vitamin B9 metabolite, 6-formyl pterin (6-FP), has been shown to bind to MR1; however; unlike 6-hydroxymethyl-8-D-ribityllumazine, 6-FP cannot activate MAIT cells (Kjer-Nielsen et al., 2012; Patel et al., 2013). In line with these findings, a previous study suggests that acetyl-6-FP, an analog of 6-FP, acts as antagonist of MR1 in the inhibition of MR1-dependent MAIT cell activation (Eckle et al., 2014). Taking into account that the kinds and levels of biosynthesized B vitamins differ among bacteria (Magnusdottir et al., 2015), these findings collectively suggest that the balance between the amount of vitamin B2 and B9 as well as the type of bacteria and its metabolism are critical factors in determining MAIT cell activation.

## Conclusion

There are many immune cells in the intestine, where biological communication between dietary components and microorganisms produces numerous kinds of metabolites. These dietary or microbial metabolites affect cell composition and function and alter energy metabolism. Accumulating evidence has revealed the importance of these changes in cell composition, function, and energy metabolism in the control of host immune responses and the subsequent incidence of inflammatory, allergic, and infectious diseases. It should be noted that not all of the results obtained in the rodent models can be directly translated to humans, because their diets and commensal bacteria differ greatly. In this context, it is important to utilize cohort studies and clinical data that include the effects of antibiotic treatment. In addition, it will be important to apply novel and/or improved technologies such as single-cell omics, whole-genome sequencing, non-target metabolomics, and bioinformatics in future research. Understanding the integrated mechanisms by which diet, microbiota, and metabolites influence the function of the immune system would be an interesting topic for future study that could provide new strategies for the control of immune and infectious diseases.

## Author Contributions

All authors listed have made a substantial, direct and intellectual contribution to the work, and approved it for publication.

## Conflict of Interest Statement

The authors declare that the research was conducted in the absence of any commercial or financial relationships that could be construed as a potential conflict of interest.

## Abbreviations

AA: arachidonic acid
DC: dendritic cell
DHA: docosahexaenoic acid
EPA: eicosapentaenoic acid
FAO: fatty acid oxidation
FP: formylpterin
GPR: G-protein-coupled receptor
IEC: intestinal epithelial cell
IL: interleukin
LCFA: long-chain fatty acid
MAIT: mucosal-associated invariant T cell
MR1: MHC-like protein 1
PD1: Protectin D1
PPAR: peroxisome proliferator-activated receptor
Rv: resolving
SCFA: short-chain fatty acid
TCA: tricarboxylic acid
TLR: toll-like receptor
TNF: tumor necrosis factor
TX: Thromboxane
